# A three‐dimensional method for morphological analysis and flow velocity estimation in microvasculature on‐a‐chip

**DOI:** 10.1002/btm2.10557

**Published:** 2023-06-11

**Authors:** Alberto Rota, Luca Possenti, Giovanni S. Offeddu, Martina Senesi, Adelaide Stucchi, Irene Venturelli, Tiziana Rancati, Paolo Zunino, Roger D. Kamm, Maria Laura Costantino

**Affiliations:** ^1^ LaBS, Chemistry, Materials, and Chemical Engineering "Giulio Natta" Department Politecnico di Milano Milan Italy; ^2^ Data Science Unit, Department of Epidemiology and Data Science Fondazione IRCCS Istituto Nazionale dei Tumori Milan Italy; ^3^ Department of Biological Engineering Massachusetts Institute of Technology Cambridge Massachusetts USA; ^4^ MOX, Department of Mathematics Politecnico di Milano Milan Italy

**Keywords:** 3D computational analysis, deep learning, network morphology, segmentation, vasculature‐on‐a‐chip

## Abstract

Three‐dimensional (3D) imaging techniques (e.g., confocal microscopy) are commonly used to visualize in vitro models, especially microvasculature on‐a‐chip. Conversely, 3D analysis is not the standard method to extract quantitative information from those models. We developed the μVES algorithm to analyze vascularized in vitro models leveraging 3D data. It computes morphological parameters (geometry, diameter, length, tortuosity, eccentricity) and intravascular flow velocity. μVES application to microfluidic vascularized in vitro models shows that they successfully replicate functional features of the microvasculature in vivo in terms of intravascular fluid flow velocity. However, wall shear stress is lower compared to in vivo references. The morphological analysis also highlights the model's physiological similarities (vessel length and tortuosity) and shortcomings (vessel radius and surface‐over‐volume ratio). The addition of the third dimension in our analysis produced significant differences in the metrics assessed compared to 2D estimations. It enabled the computation of new indices, such as vessel eccentricity. These μVES capabilities can find application in analyses of different in vitro vascular models, as well as in vivo and ex vivo microvasculature.

## 1 INTRODUCTION

Microfluidic models of the human microvasculature, also called microvasculature‐on‐a‐chip, have been used in the last decade to study several physiological and pathological phenomena involving the microvasculature and its microenvironment.^1^, ^2^, ^3^, ^4^ These investigations have included angiogenesis,^5^ organoid vascularization,^6^, ^7^ tumor cell extravasation,^8^, ^9^ immune cells extravasation/recruitment,^10^ tissue‐specific modeling,^11^, ^12^ and vascular wall properties and their effect on drug delivery.^1^, ^9^ One of the strengths of such in vitro modeling approach is an increased physiological relevance due to the ability to include human‐derived cells. In addition, those models' 3D vascular architecture results in a better microenvironment replica than classical 2D cell culture techniques.^13^ Further physiological relevance of the models can be achieved by including additional cell types, biochemical compounds, and mechanical stimuli through direct access to the microfluidic chip.^7^, ^14^ Most importantly, perfusion of the microvascular models can be achieved through a pressure difference at the network's ends.^15^ Furthermore, even a transmural pressure difference can be modeled, resulting in fluid flow across the vascular endothelium^16^ or mechanical characterization.^17^

To analyze these microvasculature‐on‐a‐chip models and to observe the microvascular network under different conditions, researchers usually adopt image‐based techniques, obtaining the images from epifluorescence or confocal microscopes.^1^, ^3^, ^4^, ^18^ However, even if the latter enables a 3D analysis, many studies usually rely on 2D‐based elaboration methods. This is particularly the case for morphological analyses, in which the microvascular networks are evaluated in vessel radii, length, and branching. Besides manual analysis, several algorithms have been proposed in the literature to tackle this problem. The AngioTool algorithm was developed to analyze the vasculogenic potential of endothelial cells and fibroblasts.^19^ This Matlab‐based code relies on 2D images and provides the radius, length, and number of junctions, even in a nonuniform image illumination. The VESGEN2D method starts from 2D binary images and is based on skeletonization and distance mapping.^20^ Besides the radius, length, and the number of junctions, it also provides the vessel density and the fractal dimension. The RAVE code analyzes 2D images with similar methods to obtain radii distribution and the vessel density as length or area ratio.^21^ Finally, the REAVER method was recently proposed.^22^ It is based on segmentation and skeletonization and provides the vessels' length, area, average diameter, and the number of junctions. The authors showed that REAVER is more accurate than the other algorithms while maintaining a reasonable computational time.

Few 3D algorithms have been proposed in the literature as applied to in vivo images.^23^, ^24^, ^25^ They are based on similar steps, segmentation, and skeletonization, leveraging distance mapping to compute different network features. Furthermore, they take advantage of the third dimension considering a volumetric vascular density. In addition, the method can provide the fractal dimension, the number of segments, vessel length, diameter, and tortuosity.^23^ These methods have shown that the third dimension can add important information to the analysis, more properly evaluating the 3D system. The circularity of the vessel cross‐section represents a clear example of how the 3D information might be used to enhance the characterization of the model. For this reason, we have developed the micro‐Vasculature Evaluation System (μVES), an algorithm capable of analyzing the morphology of in vitro microvasculature and estimating intravascular fluid flow velocity under a given pressure difference. We further show how this tool can be used to evaluate the physiological relevance of microvasculature‐on‐a‐chip models.

## 2 RESULTS AND DISCUSSION

First, we present the validation of the algorithm, along with the test of its extended 3D capability. Second, 2D and 3D analyses are compared for the same microvasculature on‐a‐chip model. Finally, we show how the method can be used to evaluate the physiological relevance of that model.

### 2.1 Algorithm validation

Established state‐of‐the‐art methods to analyze microvascular morphology rely on 2D images from fluorescence microscopy.^1^ Therefore, we tested a simplified μVES algorithm, μVES2D, that works with this kind of data for validation purposes. However, not all the output data are available in this version, such as the vessel eccentricity, which requires 3D data. Results of the algorithm are compared with a manual analysis (line‐based measures with ImageJ) and the analysis software REAVER, which provides accurate results^22^ and shares the μVES2D Matlab environment.

**FIGURE 1:**
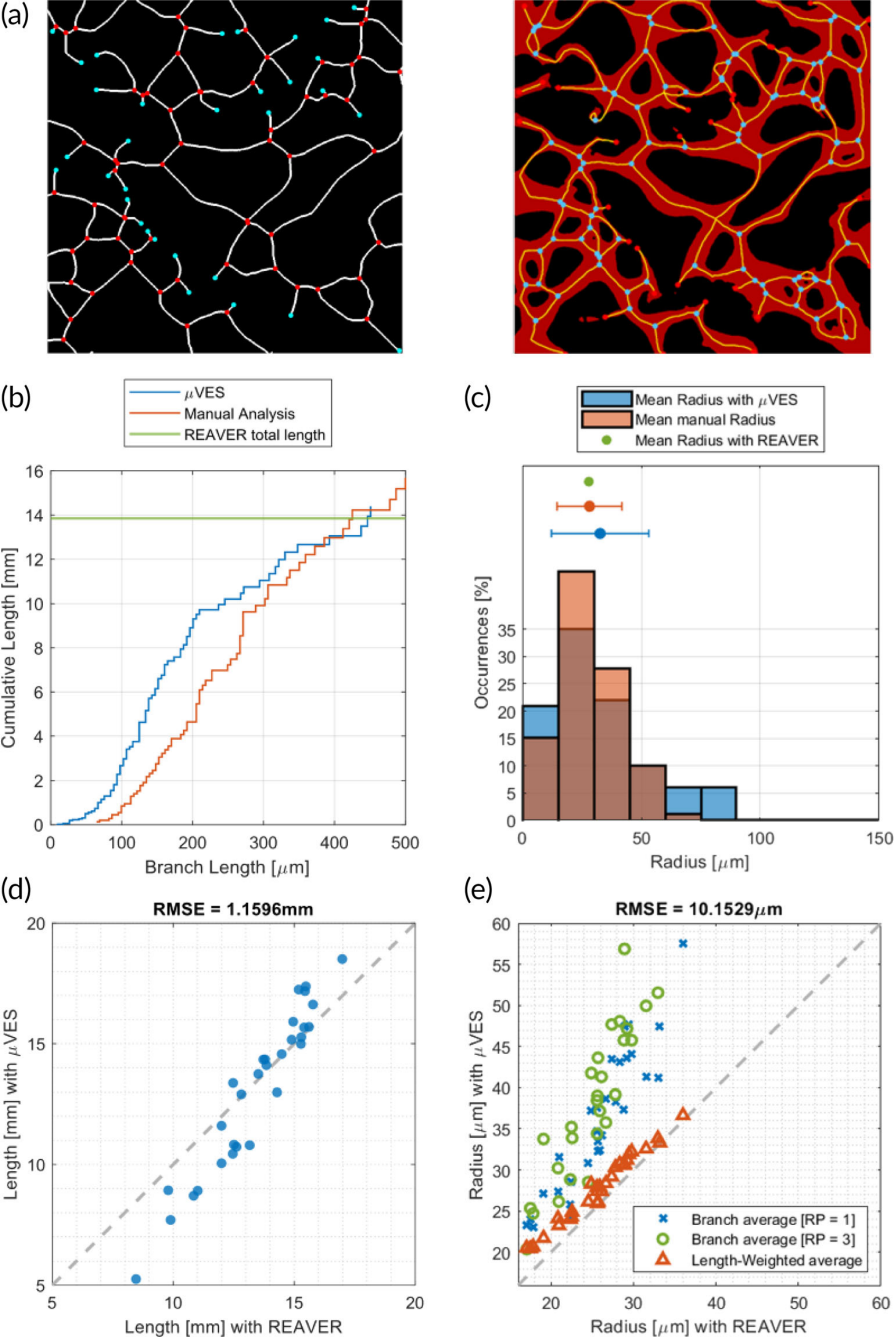
2D algorithm validation against REAVER code^22^ and manual analysis. (a) Single image comparison of the skeleton (centerlines of the vessels and junctions) with REAVER (left) and μVES code (right). The network is also presented in the μVES image for skeleton accuracy interpretation. (b) Evaluation of the radius distribution in the image and (c) the length of the network using the three methods. Agreement between the radius value (d) and the total length (e) based on the analysis of 30 images with both REAVER and μVES.

After a single network analysis (Figure 1a), the radius comparison shows a good qualitative agreement considering the data distribution (Figure 1c) with a slight difference in average values (REAVER: 27.8 μm, Manual analysis: 29.6 ± 12.1 μm, μVES2D [*n*_*r*_ = 1]: 32.6 ± 20.5 μm). Relying on single‐image data, the main difference between manual analysis and the μVES algorithm concerns a few large vessels (100–150 μm) not captured in the manual analysis. Furthermore, algorithms were tested on 30 different images (Figure 1e). The mean radius for a network has been computed with *n*_*r*_ equal to 1 and 3, and with a weighted average on the vessel length. The REAVER code also uses the latter technique, not relying on network branching. When using this method to compute the radius, μVES and REAVER results are in good agreement (Figure 1e; Bland–Altman test—Bias = 1.94 μm, biasSD = 0.88 μm, LOA = 0.2107–3.6688 μm). However, the *n*_*r*_ = 1 or *n*_*r*_ = 3 method results in a different mean value due to the different averaging techniques.

The length analysis on a single image reports similar values for the total network length (REAVER: 13.9 mm, Manual analysis: 15.7 mm, μVES2D: 14.4 mm, Figure 1b). Furthermore, the cumulative length curve is similar when comparing μVES2D and the manual analysis, with smaller vessels identified better by the algorithm. We excluded the manual analysis to test the length measure on multiple networks and focused on the total network length (Figure 1d). Good agreement between the two algorithms can be observed with data close to the diagonal (Bland–Altman test—Bias = 0.33 mm, biasSD = 1.43 mm, and LOA = −2.5‐3.1 mm). However, in the μVES2D method, vessel length is computed relying on the interpolation of the coordinates, whereas in the REAVER method by counting the skeleton pixels. This difference may contribute to the scatter of the plot.

Comprehensively, these data validate the μVES method by leveraging its 2D version, enabling comparison with available algorithms.

### 2.2 From 2D to 3D analyses

The peculiarity of the μVES method is its capability to analyze 3D data. This kind of data can be readily available from confocal microscopy. With the third dimension added in, it is important to discuss two main microvascular network images. The first relies on a fluorescent marker of ECs, for example, cell membrane, glycocalyx, and GFP produced by cells. The second is based on the perfusion of the network with a fluorescent probe. Even if the 2D projection of these two cases may result similar, they profoundly differ in the 3D data as the first is comparable to the lateral area of vessels while the second represents their internal volume. The μVES method uses the second kind of data, thus excluding the network's nonperfusable part, which cannot be reached by the fluorescent probe.

**FIGURE 2:**
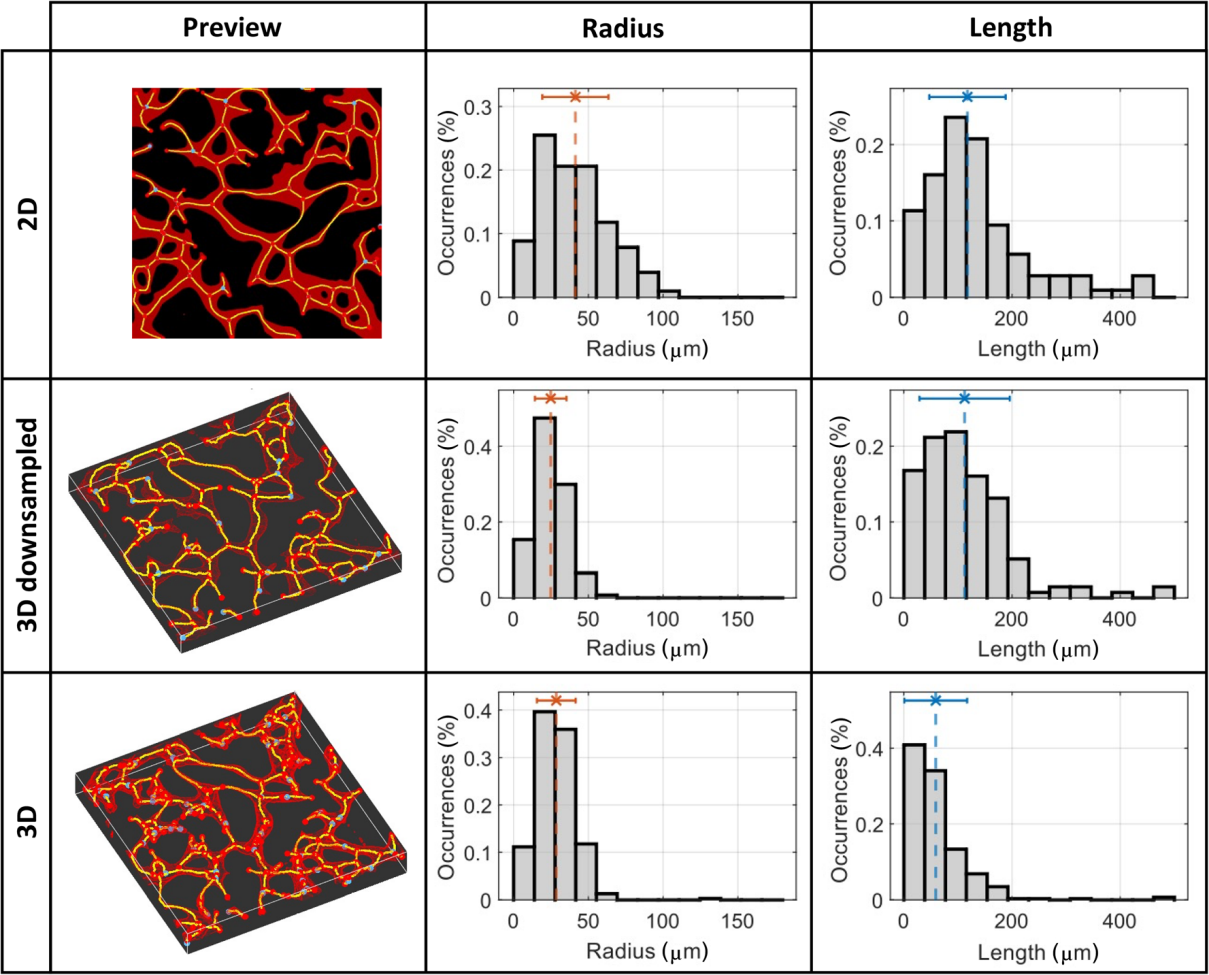
Comparison of the network reconstruction and the vessel radius and length distributions for the three types of analysis (2D, 3D DWS, 3D). The dotted lines on the graphs show average values. DWS, downsampling.

We selected a single network and analyzed it with both the 2D (on the z‐projection) and the full version of the μVES algorithm (Figure 2). The difference in the average radius is significant (2D: 41.3 ± 22.1 μm, 3D: 28.5 ± 13.0 μm), and the two distributions look very different. This large discrepancy is due to an elliptical cross‐sectional area of the microvessels.^26^ The low eccentricity value confirms this feature of the vessels in the model assessed for these vessels (Figure S1). This parameter describes an essential feature of the in vitro microvascular networks, typically characterized by ellipsoidal cross‐sections with the major axis oriented horizontally (Figure S1). Due to this phenomenon, the 2D analysis of the vessel radius returns incomplete information, losing essential knowledge on the vertical direction.

Therefore, we recommend adopting the 3D analysis when accurate information is required.

The length of the network is also different in the two analyses, both as the total length of the network (2D: 17.6 mm, 3D: 19.0 mm) and distribution (Figure 2). As expected, the total length of the 2D projection is smaller than the 3D one, given that the third dimension is removed. On the other hand, the distribution is shifted toward shorter vessels due to the greater number of small vessels reconstructed, especially close to the junctions. We point out that a threshold can be set to cut vessels shorter than a value. Such operation indeed affects the distribution shown and may generate a variation of the total length.

Finally, the 3D analysis allows the computation of the lateral area (Figure S1) and the S/V parameter, namely the ratio between the vascular surface, the area of the microvascular network available for exchanges, and the extra‐vascular volume. This parameter is essential when analyzing mass transport, as it directly determines fluid and solute exchanges.^27^ Importantly, this parameter again cannot be estimated from 2D analyses, mainly due to the noncircular cross‐section of the vessels, further showcasing the advantages of 3D analysis.

### 2.3 Computational cost and downsampling

3D analyses extract more information from the images, but these methods have a greater computational demand compared to 2D‐based ones. To reduce such requirements, we considered downsampled analyses (Figure 2). For a reference image, computational time was reduced from 13.53 to 1.17 min (−91.3%) when including the downsampling. Results regarding the vessel radius distribution are similar to the 3D ones and consequently dissimilar to the 2D ones. Indeed, the vessel cross‐section anisotropy is successfully determined even with a downsampled volume. Conversely, the results regarding vessel length agree with the 2D analysis. This phenomenon is related to the resolution loss that follows from the downsampling operation, which cuts off shorter vessels, as seen in the three graphs in Figure 2. Therefore, the 3D downsampled analysis allows the complete volumetric image analysis, reducing the computational cost, and the analysis resolution.

### 2.4 Deep learning approach for segmentation

**FIGURE 3:**
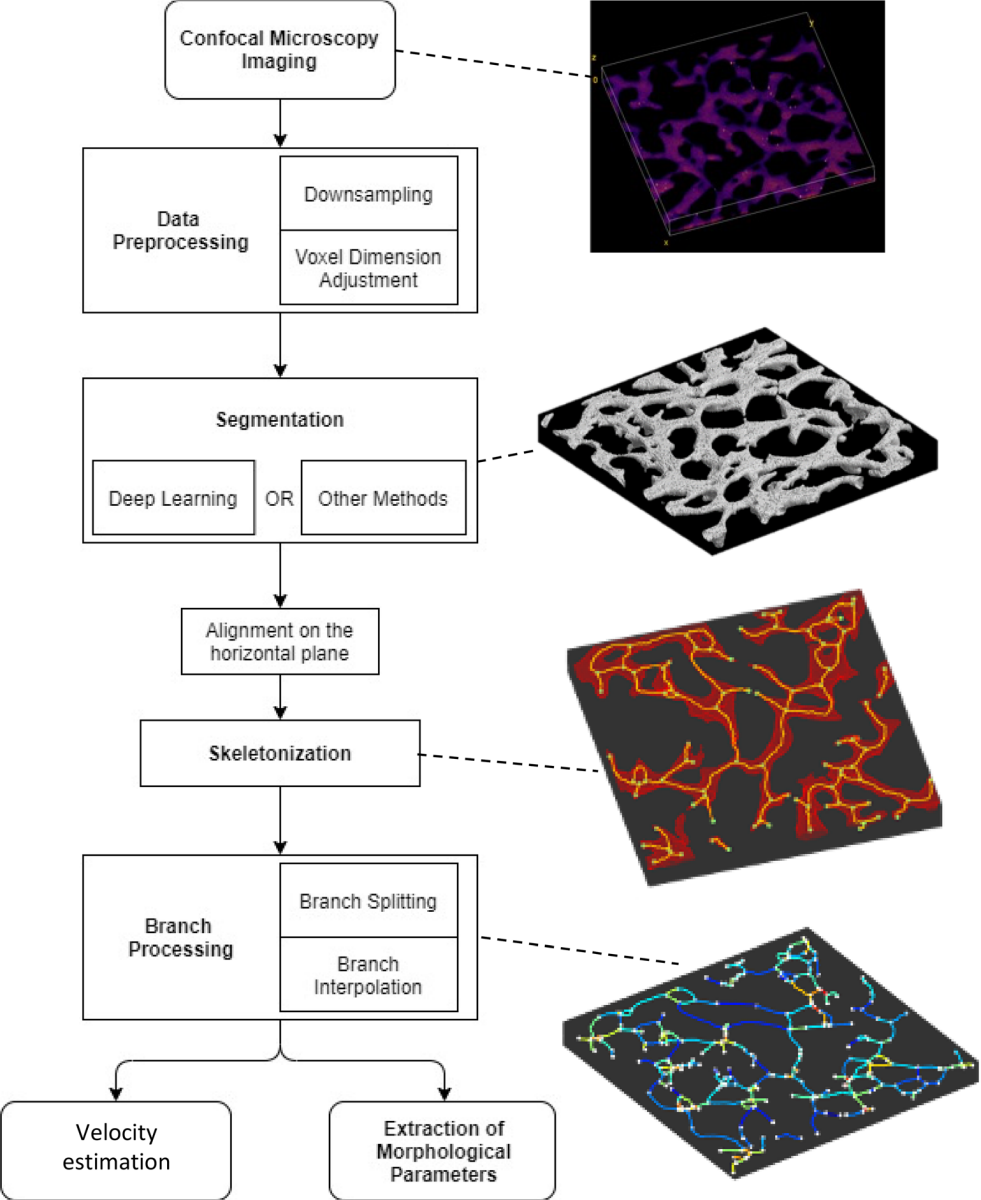
Scheme of the μVES algorithm starting from confocal imaging to the outputs: (i) image acquisition, (ii) data preprocessing including possible downsampling operations and volumetric interpolation, (iii) network segmentation with active contour method or deep learning‐based classification, (iv) vertical alignment, (v) skeletonization, (vi) branch processing identifying different branches in the network and interpolating the spatial coordinates, (vii) descriptive metrics computation, and (viii) velocity estimation. The four images on the right depict key steps of the algorithm, which are identified by a dotted line.

**FIGURE 4:**
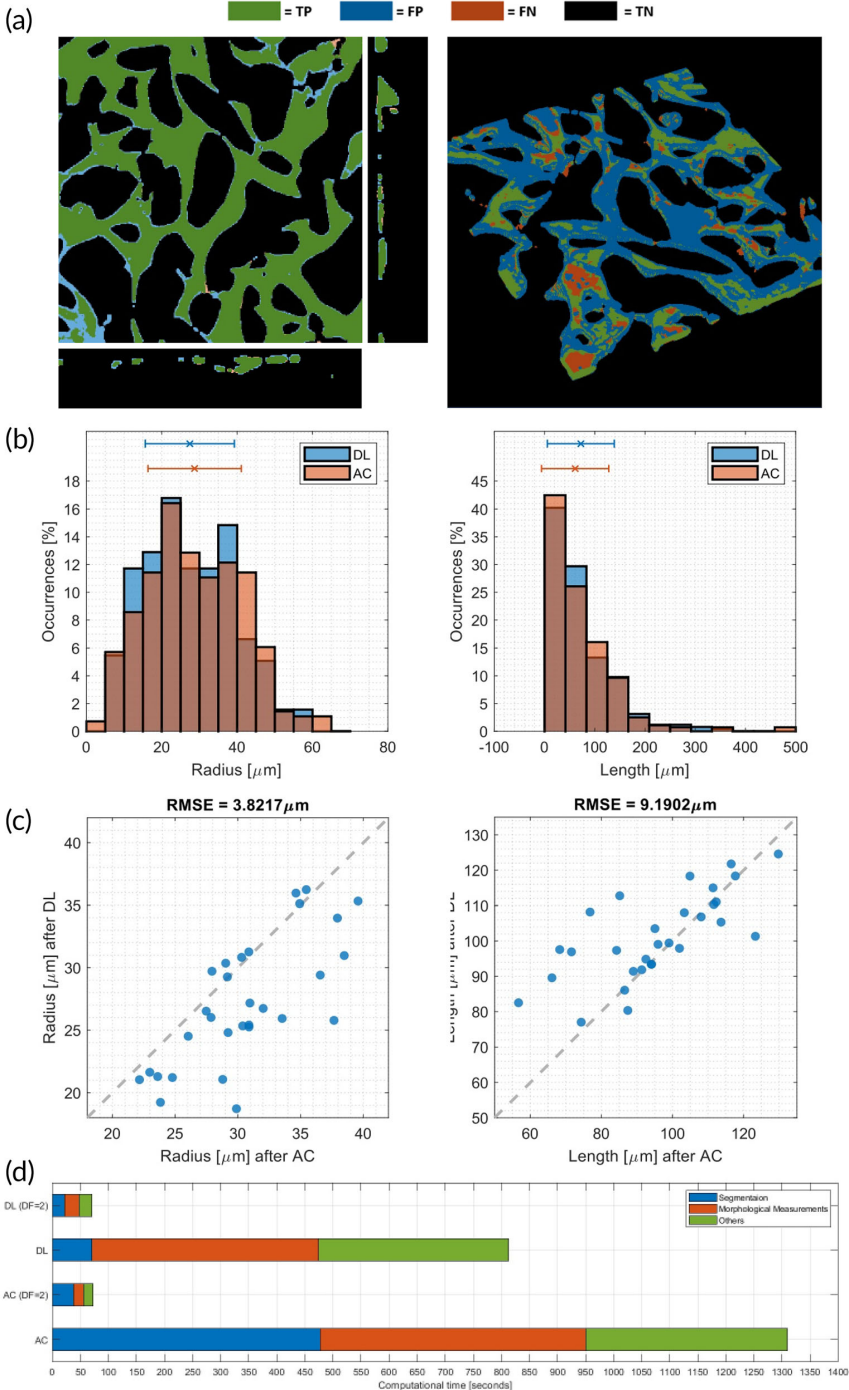
Deep learning segmentation versus Active contour segmentation on a single network (a) showing true positive (TP), false positive (FP), false‐negative (FN), and true negative (black). Radius and length comparison over the validation set (b) and their distribution on a single image (c). The computational time (d) was compared using the two methods with different downsampling factors (DF).

Besides the downsampling operation, we applied a deep learning approach to reduce computational requirements. This method substitutes the active contour algorithm in the workflow (Figure 3), which is one of the most demanding operations of the analysis (Figure 4). Based on 56 augmented images, the training process ended with a validation binary accuracy of 87.52%, a weighted Jaccard index of 73.20%, and a Matthews Correlation Coefficient of 69.38%. Additionally, discrete noise‐rejection capabilities are highlighted by a Specificity of 93.16%. The application of this method to a single image reveals the localization of the false positive and the false negative pixels to the network edges. Conversely, the inner part of the vessels is characterized by the strong presence of true positive pixels (Figure 4a). Being particularly interested in extracting descriptive parameters, we analyzed the effect of this alternative approach on two metrics, vessel length and radius, rather than on voxel‐wise segmentation. On a single image analysis, the distribution of both the variables is similar (Figure 4b), also reporting similar average values (deep learning: *r* = 28.12 ± 11.29 μm, *L* = 72.13 ± 66.67 μm; active contour: *r* = 28.98 ± 11.83 μm, *L* = 70.32 ± 66.95 μm) with no statistical difference (*p*‐values 0.39 and 0.62 for radius and length, respectively). When applying the methods on multiple networks, we obtained similar results (Figure 4c, Bland–Altman test: radius—Bias = 3.39 μm, biasSD = 3.64 μm, LOA = −10.5–3.8 μm; Bland–Altman test: length—Bias = 5.7 μm, biasSD = 12.7 μm, LOA = −30.5–19.1 μm). The computational cost reduction for segmentation depends on the sample's dimension (Figure 4d) and, therefore, on the downsampling factor (factor 2: −46.12%, factor 1: −78.47%).

### 2.5 Application to microvasculature‐on‐a‐chip

We applied the μVES algorithm to a set of 30 images depicting microvascular networks on a chip. First, the average capillary length in the microfluidic chips was 102.9 ± 76.1 μm. This result agrees with literature reporting capillary vessel lengths from tens to hundreds of microns.^23^, ^25^, ^28^, ^29^ The average vessel diameter among all the samples was 31.4 ± 16.2 μm, consistent with data previously reported for this application.^1^ Conversely, this result is still somewhat far from the capillary diameter reported in vivo, which approaches the size of a single red blood cell, 6–10 μm.^30^ In a similar analysis based on mice coronary capillary networks, Nicolas and Roux reported a range spanning from 2 to 14 μm in vessel diameter, in which the 4–6 μm interval is the most frequent.^23^

The same authors also studied vessel tortuosity. These data are rarely included in the studies, and when considered, it often involves different definitions. As we used the same definition as Nicolas and Roux, we can directly compare our results. They reported a vessel tortuosity index in vivo from 1 (perfectly straight vessel) to 3, with a distribution skewed toward 1. In our dataset (in vitro), the tortuosity index ranged from 1 to 2.35 (average: 1.18). When comparing the in vitro morphological parameters with in vivo data, we must consider the restriction of the model in the z‐direction, which affects the microvascular network geometry. Anyway, in vitro tortuosity data are consistent with the available in vivo data. This observation is interesting, considering that no flow conditioning has been applied during EC culture. Indeed, adding mechanical stimuli during culture (i.e., from intravascular flow) seems unnecessary to replicate the vessel tortuosity.

A further important feature is the surface of the network available per unit of volume (S/V), which is particularly meaningful when studying mass exchanges and microvascular wall properties. S/V differs from tissue to tissue and from physiological to pathological cases. As reference values, we report 7 mm^2^/mm^3^ as an average for healthy tissue,^31^ whereas hyper‐vascularized tumors can reach up to 26 mm^2^/mm^3^.^32^ Our in vitro samples present an S/V = 4.5 ± 1.3 mm^2^/mm^3^, similar to previously reported data.^33^ Such value marks a difference between the in vitro networks and the in vivo reference. Anyway, this last result is undoubtedly affected by the planar geometry of the microvasculature on a chip, whereas in vivo vasculature might have a more 3D structure. In vitro models with a more 3D design have been proposed,^34^ even if perfusion of the network has not been achieved.

As described above, the μVES algorithm also allows us to compute the vessels' eccentricity. Generally, the blood vessel cross‐section is considered circular in vivo, with eccentricity close to 1 (perfect circle). However, in vitro data show a vessel eccentricity equal to 0.833 ± 0.125, implying a noncircular shape (Figure S1) as the in vitro vessel cross‐section approaches an ellipse with the major axis oriented horizontally according to previously reported data.^26^

Summarizing, the different analyses showed how in vitro microvasculature on‐a‐chip represents a physiologically relevant model, even if they can be improved in some features. Differences between in vivo and in vitro microvascular networks might arise from changes in the experimental protocols used,^35^, ^36^ resulting in a better or worse replica of the characteristics analyzed.

### 2.6 Velocity estimation

**FIGURE 5:**
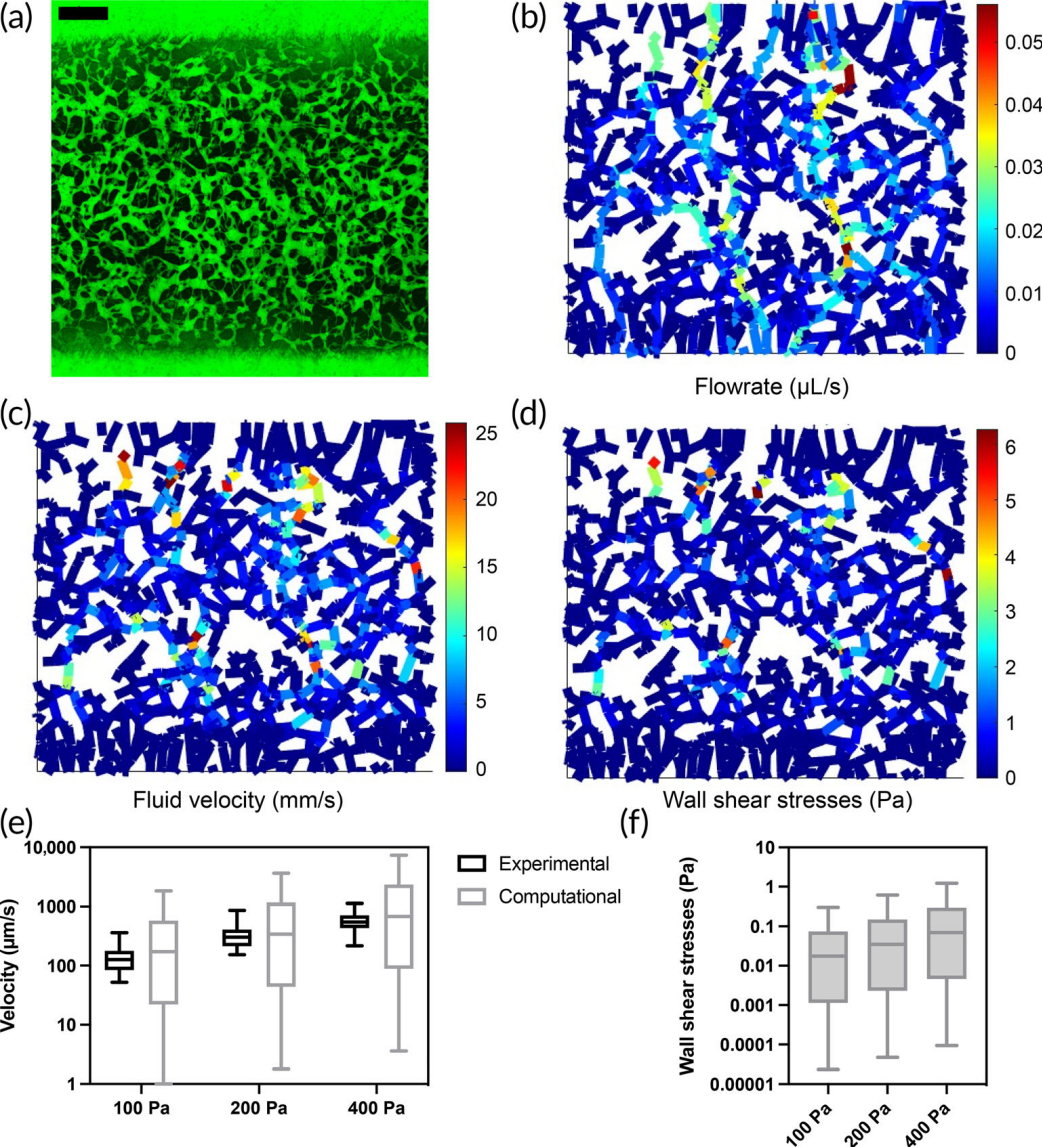
Functional velocity evaluation. The raw image of the network—perfused with FITC labeled IgG (a)—was used to generate flow rate (b), velocity (c), and wall shear stresses (d) map. The distribution of variables is shown for the velocity (e)—against in vitro measures—and for the wall shear stresses (f). Scale bar in (a) is 500 μm.

An essential trait of microvasculature‐on‐a‐chip models is the capability to perfuse the system. This enables the modeling of flow‐related phenomena^15^, ^16^ from the mechanical action of the flow on endothelial cells^37^, ^38^ to the pressure‐driven exchange through the microvascular wall. To evaluate the model under this point, we perfused the network by applying three different pressure gradients (100, 200, and 400 Pa). The experimental measure revealed median velocities of 126.4, 303.1, and 545.5 μm/s, respectively. Given the not normally distributed data, we consider the median values to compare the velocity distribution referring to a set of vessels. Furthermore, the average velocity (155, 354, and 596 μm/s) is in the same order of magnitude as the median. The μVES algorithm further allows the computation of fluid flow rates in the microvascular networks analyzed. Here, we applied the algorithm to evaluate a large chip image with dimensions 3.67 × 3.81 × 0.15 mm^3^ (Downsampling factor = 2), which depicted a portion of the central channel from side to side (Figure 5). Preferred flow paths are evident in the flow rate plot yielded by the μVES algorithm, showing heterogeneity of fluid flow within the network. The μVES algorithm results reasonably agree with the experimental data (Figure 5), increasing linearly with the applied pressure difference (median values 171.3, 342.6, and 685.2 μm/s). The small difference between experimental and computational values has different possible sources. First, practical measures cannot be performed over a wide area of the chip, as can be quickly done with computational methods. Second, these experimental measurements are performed using bead velocity as a proxy for fluid flow velocity, although adhesive interactions between beads and the vascular wall are common, resulting in lower velocities measured. Third, modeling approximations play a role, from geometry reconstruction to the simplified flow model (assuming circular vessels). Fourth, even if it represents a minor contribution, experimental data are produced by projection on a plane, therefore losing the z‐direction, as detailed in the Section 3.

Further, based on these results, we used the μVES algorithm to estimate the wall shear stress (WSS) for each branch, resulting in a median value of 1.7 × 10^−2^, 3.5 × 10^−2^, and 7.0 × 10^−2^ Pa.

The flow velocity results agree with the reported values from the literature. Indeed, blood velocity values span from hundreds of μm/s to several mm/s,^39^, ^40^, ^41^ with 1 mm/s accepted as an average reference in capillaries.^14^ On the other hand, the WSS results appear lower than the literature values, which reach a few Pa.^42^ This is consistent with previous results in terms of velocity and microvascular radii. Indeed, in vitro vascular radii are more similar to arterioles or venules. Therefore, in the in vitro system, a physiological flow velocity and WSS cannot be achieved simultaneously (see the equation for WSS, $\tau_{i}$). Instead, the pressure drop can be set to reproduce a physiological velocity with lower WSS or a physiological WSS with velocities greater than the physiological reference. Further, the ellipticity of the cross‐section was not considered in the velocity and WSS computation, even if it has a minor effect when considering steady flow.^43^ Finally, red blood cells are not present in the in vitro model, and their presence significantly impacts the WSSs.^44^ The μVES algorithm estimates WSSs assuming a Newtonian fluid, which correctly describes the in vitro model. Under these conditions, the algorithm can be used to estimate the pressure drop required to reach a desired level of WSS. This assessment attests to the capabilities of the μVES algorithm to go beyond morphological analyses of microvascular networks, including evaluations of microvascular function.

As a final remark, we computed how the uncertainty in the radius affects the velocity and the WSS results. We first show a theoretical analysis on a single vessel, leveraging uncertainty propagation. Considering dr≈1.6μm as the uncertainty on the radius, we can estimate its effect on the uncertainty on the velocity (dv) and the WSS (dWSS) estimation:

$$\mathrm{dv}=\frac{∆p\;}{8\;\mu \;l}\;2r\;\mathrm{dr}\approx 107\;\frac{\mathrm{\mu m}}{s}.$$

$$\text{dWSS}=\frac{∆p}{2\;l}\;\mathrm{dr}\approx 14\;\mathrm{mPa}.$$

We also performed a computational analysis on a simplified version of a vascular network, including eight vessels. The uncertainty effect computed referring to the median values over the network is still comparable to the theoretical estimates provided (Figure S2).

## 3 MATERIALS AND METHODS

The μVES algorithm comprises eight different steps: (i) image acquisition, (ii) data preprocessing, (iii) network segmentation, (iv) vertical alignment, (v) skeletonization, (vi) branch processing, (vii) descriptive metrics computation, and (viii) velocity estimation (Figure 3). The method* was coded using MATLAB^45^ (The MathWorks Inc., 2021). The following paragraphs describe each step of the process.

### 3.1 Microvascular network generation and image acquisition

The microvascular networks are generated within microfluidic chips (central channel dimension: 3 × 10 × 0.5 mm^3^) by vasculogenic self‐assembly in a fibrin gel, exactly as described previously.^16^ Briefly, we seeded GFP HUVECs (Angio‐Proteomie, 6 Mcells mL^−1^) with human lung fibroblasts (Lonza, 2 Mcells mL^−1^) in the central channel (width = 3 mm) of a three‐channel microfluidic chip and cultured the system through daily media changes in the side channels for 7 days. After microvascular network maturation on day 7, TRITC fluorescent dextran is inserted in the chip to assess the vascular network perfusion by confocal imaging (Olympus FV1000 confocal microscope with a custom enclosure for temperature and atmosphere control). Images have 800 × 800 pixels resolution in the x–y direction, with 0.98 μm/pixel, and a 5 μm step in the z‐direction, resulting in a noncubic voxel.

### 3.2 Data preprocessing

First, we processed the images to obtain cubic voxels by interpolating the z‐direction (using the x‐y pixel dimension). Second, an optional downsampling operation was performed. Such an operation reduces the size of the image to be analyzed by selecting one out of *n* pixels in the three directions, reducing the number of pixels by a factor *n*,^3^ and decreasing the computational time. This step is beneficial when dealing with highly detailed images, as a reduced resolution will not affect the morphology of the network (see Section 2). Moreover, downsampling may be beneficial in terms of noise reduction. We remark that no specific procedure to handle unspecific signals (e.g., autofluorescence of other components) is performed. Therefore, these artifacts must be avoided or minimized during imaging.

### 3.3 Network segmentation

The segmentation step generates a 3D matrix of boolean data, defining the microvascular network volume. Among the available methods, we selected the “Active Contour Without Edges” algorithm^46^ due to its large‐scale use in the biomedical field.^47^, ^48^ In this algorithm, an initial rough approximation of the segmented vasculature gets iteratively refined to convergence until the final binary mask is obtained. We obtain reliable and consistent segmentations at the expense of computational time. Alternatively, we implemented a deep learning method. Such an approach is widely used to segment 2D images.^49^, ^50^, ^51^ Therefore, we modified it to handle 3D images. The method consists of a convolutional neural network based on the U‐Net model^52^ trained with a dataset of 3D images obtained from 70 samples, 80% dedicated to training and 20% to validation. Due to the scarcity of instances, the training dataset has been submitted to a data‐augmentation procedure. The augmenting transformations comprise the randomized combination of horizontal and vertical flipping, rotations, zooms, and translations with mirror padding. The neural network works on sections of the input matrix (128 × 128 × 8 pixels), individually segmented and arranged back into the initial position to recompose the whole volume. This operation reduces the time required for the training process and, alongside the data augmentation process (~20 patches extracted from each image), allows us to obtain a rich dataset from a relatively low amount of 3D images.

### 3.4 Vertical alignment

Due to possible small misalignments of the microfluidic chip and the microscope, the microvascular network may be artificially sloped with respect to the horizontal plane. Therefore, the network is aligned by considering an interpolating planar surface and applying the mean squared error minimization method.

### 3.5 Skeletonization

We reduced the segmented network to its centerline, obtaining single‐pixel‐wide branches. We employed the well‐established Lee–Kashyap–Chu implementation.^53^

### 3.6 Branch processing

Branches are identified as a set of pixels in the skeleton separated by either ramifications or terminal voxels. Then, coordinates for each branch are extracted by applying a graph‐theory‐based approach^54^ and interpolated with a spline curve to obtain a continuous branch‐wise representation of the skeleton.

### 3.7 Descriptive metrics computation

The morphological parameters are computed starting from the skeleton and the 3D data. First, the vessel's length is approximated with the sum of Euclidean distances for adjacent skeleton points. Tortuosity is then defined for each branch by the start‐to‐end Euclidean distance over the length.

Other metrics are computed, complementing the skeleton information with the 3D data. The radius of each branch is evaluated at *n*_*r*_ equidistant points on the arc‐length coordinate system. For each point, the binary volume is sliced perpendicular to the vessel, and the radius is computed as half of the equivalent diameter of the vascular cross‐section. Eccentricity is estimated by approximating ellipses on the edge pixels of the same cross‐sections:

e=1nr∑r1−brar2

where *a* is the major axis, and *b* is the minor axis. Lastly, the lateral area of each branch is computed leveraging the *n*_*r*_ radii and the length of each vessel segment.

### 3.8 Velocity measures and estimation

Experimental flow velocity measures are based on 2 μm fluorescent microspheres (R&D Systems) perfused through the vascular network under a hydrostatic pressure difference between the two side channels of the microfluidic chip. The networks are primarily oriented in the xy plane since they have a height of a few hundred μm (x–y area: 3 × 10 mm^2^). Velocity measurements for single beads were performed on a Nikon Eclipse Ti microscope with a 4× objective exactly as done previously^55^ by measuring the track length over the image acquisition time using the software ImageJ. As before, the assumption was made that most bead movement takes place on the xy‐plane, resulting in the loss of the z‐direction component in the analysis of the overall bead velocity. Computationally, the analysis is based on the skeleton computed as above, along with the geometrical data of each vessel. Neglecting fluid filtration, which is often minimal compared to the intravascular flow rate, we developed here an algorithm to solve the algebraic system of Poiseuille flow and mass conservation equations:

$$\left\{\begin{array}{c} p_{\text{in},i}-p_{\text{out},i}=R_{i}\;Q_{i}\;\text{with}\;i=1,\ldots ,n_{\text{branch}}\\ \sum_{i\in j}Q_{j}=0\;\text{with}\;j=1,\ldots ,n_{\text{nodes}} \end{array}\right.$$

where $R_{i}=\frac{128\;\mu \;L_{i}}{\pi \;D_{i}^{4}}$ is the hydraulic resistance. The system has a total of $\left(n_{\text{branch}}+n_{\text{nodes}}\right)$ equations. Results describe the flow rate for each branch (*Q*_*i*_) and the pressure at each node (*p*_*j*_). Starting with these results, we evaluated each vessel's velocity (*v*_*i*_), dividing the flow rate by the cross‐section. Furthermore, we estimated the WSS, enforcing the same assumptions required for Poiseuille flow:

$$\tau_{i}=\frac{4\;\mu \;v_{i}}{r_{i}}$$

Vessels close to the boundary region of the gel have a more complex pattern, and they are usually not considered in the velocity measures. Therefore, we excluded them and reported all the other branches in the central portion of the image (1.78 × 2.35 mm^2^). Then, we defined constant pressure at the two sides, mimicking the pressure drop applied in the experimental setups.

Besides the straightforward velocity computation based on Poiseuille equations, a more advanced algorithm has been proposed.^56^ Such a computational model solves a fully coupled 3D–1D simulation, accounting for fluid filtration from the network. Additionally, this method can account for the red blood cells effect, namely the Fahraeus–Lindqvist and Zweifach–Fung effects,^30^ which are not included in the simplified Poiseuille estimation. The present algorithm provides the input files required for such a complete simulation.

## 4 CONCLUSION

We have presented μVES, a 3D image analysis algorithm for microvascular networks that can be used to assess the physiological relevance of microvasculature‐on‐a‐chip models in terms of morphology and functional perfusion capacity. The in vitro model successfully replicated intravascular fluid flow velocity in the microvasculature in vivo. The associated WSS are still lower than in vivo, but closer values can be achieved by increasing the applied pressure drop according to the model prediction. On the other hand, some of the morphological features of the in vitro microvascular networks do not fully represent the microvasculature in vivo. Particularly, the radius of the vessels is larger in the in vitro model, and the surface‐to‐volume ratio is still lower than in the in vivo reference. Conversely, the tortuosity and length of the in vitro vessels are comparable, achieving a satisfactory replica.

This work has shown the importance of a 3D analysis method for in vitro microvascular models, which provides information not available with a classical 2D approach and significantly impacts the computed metrics. The μVES method for morphological and functional 3D analyses offers significant improvements over previous 2D methods. The algorithm may find applications in the analysis of other in vitro models and in vivo microvasculature, comprising imaging methods that reveal the internal vessel lumen.

## AUTHOR CONTRIBUTIONS

**Alberto Rota:** Conceptualization (equal); data curation (equal); formal analysis (equal); investigation (equal); methodology (equal); project administration (equal); software (equal); validation (equal); visualization (equal); writing – original draft (equal); writing – review and editing (equal). **Luca Possenti:** Conceptualization (lead); data curation (equal); formal analysis (equal); funding acquisition (equal); investigation (equal); methodology (equal); project administration (equal); software (equal); validation (equal); visualization (equal); writing – original draft (lead); writing – review and editing (equal). **Giovanni Stefano Offeddu:** Conceptualization (equal); data curation (equal); formal analysis (equal); funding acquisition (equal); investigation (equal); methodology (equal); project administration (equal); validation (equal); visualization (equal); writing – original draft (equal); writing – review and editing (equal). **Martina Senesi:** Data curation (supporting); investigation (equal); writing – review and editing (equal). **Adelaide Stucchi:** Data curation (supporting); investigation (equal); writing – review and editing (equal). **Irene Venturelli:** Data curation (supporting); investigation (equal); writing – review and editing (equal). **Tiziana Rancati:** Funding acquisition (equal); supervision (equal); writing – review and editing (equal). **Paolo Zunino:** Supervision (equal); writing – review and editing (equal). **Roger Kamm:** Funding acquisition (equal); resources (equal); supervision (equal); writing – review and editing (equal). **Maria Laura Costantino:** Funding acquisition (equal); resources (equal); supervision (equal); writing – review and editing (equal).

## CONFLICT OF INTEREST STATEMENT

Roger D. Kamm is a co‐founder of AIM Biotech that markets microfluidic systems for 3D culture and receives research support from Amgen, Roche, Glaxo‐Smith‐Kline, and Boehringer‐Ingelheim.

## Supporting information


**Figure S1:** Orientation of the major diameter of the fitted elliptical cross‐section of the vessels on a single image (a). Cross‐section of a portion of the network obtained with deep learning (b) with true positive (TP—green), false positive (FP—blue), false‐negative (FN—red), and true negative (TN—black). (c) Eccentricity map on a network. (d) Drawings showing the difference between 2D and 3D data to estimate the network lateral area. The top view depicts 2D analysis. The mid‐level represents the lateral area computation based on the 2D radius, that is, implying a circular section. The bottom view shows results from 3D images, reporting a noncircular cross‐section.
**Figure S2:** Computational analysis of the radius uncertainty effect on velocity and WSS estimates. We analyzed a single vessel (a) and a simplified network of eight vessels (b). (c) With reference to a single vessel, we set a nominal radius of 15 μm and a radius uncertainty of 1.6 μm. We then created 106 scenarios with a radius from a normal distribution with a mean of 15 μm and a standard deviation of 1.6 μm. We fix the length (100 μm) and the pressure difference (to ensure 500 μm/s when choosing *r* = 15 μm). We report the resulting velocity and WSS distributions. Interestingly, WSSs are normally distributed (avg: 133 mPa, SD: 14 mPa), while the velocity is not (but its square root follows a Gaussian distribution). (d) Median values for radius, velocity, and WSS over the eight‐vessel network considering the same nominal radius for each vessel. Data are not normally distributed anymore. The median value variations are still comparable to the single branch case. (e) Results from the eight‐vessels network with nominal radius values randomly chosen among the permutation of the set {13, 14, 14, 15, 15, 16, 16, 17} μm. Then, we applied similar methods to compute 106 cases with 1.6 μm radius uncertainty for each vessel. Ranges of variation for median values are still comparable to the previous case.

## Data Availability

The data that support the findings of this study are available from the corresponding author upon reasonable request.

## Endnote

* The code is available at https://github.com/alberto-rota/muVES.

## References

[1] Haase K , Kamm RD . Advances in on‐chip vascularization. Regenerative Med. 2017;12(3):285‐302. doi:10.2217/rme-2016-0152 PMC5574321 28318376

[2] Kim S , Kim W , Lim S , Jeon J . Vasculature‐on‐a‐chip for in vitro disease models. Bioengineering. 2017;4(4):8. doi:10.3390/bioengineering4010008 28952486 PMC5590435

[3] Moses SR , Adorno JJ , Palmer AF , Song JW . Vessel‐on‐a‐chip models for studying microvascular physiology, transport, and function in vitro. Am J Physiol Cell Physiol. 2021;320(1):C92‐C105. doi:10.1152/ajpcell.00355.2020 33176110 PMC7846973

[4] Tronolone JJ , Jain A . Engineering new microvascular networks on‐Chip: ingredients, assembly, and best practices. Adv Funct Mater. 2021;31(14):2007199. doi:10.1002/adfm.202007199 33994903 PMC8114943

[5] Shin Y , Han S , Jeon JS , et al. Microfluidic assay for simultaneous culture of multiple cell types on surfaces or within hydrogels. Nat Protoc. 2012;7(7):1247‐1259. doi:10.1038/nprot.2012.051 22678430 PMC4035049

[6] Zhao X , Xu Z , Xiao L , et al. Review on the vascularization of organoids and organoids‐on‐a‐chip. Front Bioeng Biotechnol. 2021;9:9. doi:10.3389/fbioe.2021.637048 PMC8072266 33912545

[7] Shirure VS , Hughes CCW , George SC . Engineering vascularized organoid‐on‐a‐chip models. Annu Rev Biomed Eng. 2021;23(1):141‐167. doi:10.1146/annurev-bioeng-090120-094330 33756087

[8] Chen MB , Whisler JA , Fröse J , Yu C , Shin Y , Kamm RD . On‐chip human microvasculature assay for visualization and quantification of tumor cell extravasation dynamics. Nat Protoc. 2017;12(5):865‐880. doi:10.1038/nprot.2017.018 28358393 PMC5509465

[9] Sontheimer‐Phelps A , Hassell BA , Ingber DE . Modelling cancer in microfluidic human organs‐on‐chips. Nat Rev Cancer. 2019;19(2):65‐81. doi:10.1038/s41568-018-0104-6 30647431

[10] Parlato S , Grisanti G , Sinibaldi G , Peruzzi G , Casciola CM , Gabriele L . Tumor‐on‐a‐chip platforms to study cancer–immune system crosstalk in the era of immunotherapy. Lab Chip. 2021;21(2):234‐253. doi:10.1039/D0LC00799D 33315027

[11] Colombo MV , Bersini S , Arrigoni C , Moretti M . 3D biofabricated In vitro models of vascularized and mineralized bone tissues. Methods Mol Biol. 2022;2373:283‐296. doi:10.1007/978-1-0716-1693-2_17 34520019

[12] Ragelle H , Dernick K , Khemais S , et al. Human retinal microvasculature‐on‐a‐chip for drug discovery. Adv Healthc Mater. 2020;9(21):2001531. doi:10.1002/adhm.202001531 32975047

[13] Low LA , Mummery C , Berridge BR , Austin CP , Tagle DA . Organs‐on‐chips: into the next decade. Nat Rev Drug Discov. 2021;20(5):345‐361. doi:10.1038/s41573-020-0079-3 32913334

[14] Myers DR , Lam WA . Vascularized microfluidics and their untapped potential for discovery in diseases of the microvasculature. Annu Rev Biomed Eng. 2021;23(1):407‐432. doi:10.1146/annurev-bioeng-091520-025358 33863238 PMC8785195

[15] Hajal C , Ibrahim L , Serrano JC , Offeddu GS , Kamm RD . The effects of luminal and trans‐endothelial fluid flows on the extravasation and tissue invasion of tumor cells in a 3D in vitro microvascular platform. Biomaterials. 2021;265:120470. doi:10.1016/j.biomaterials.2020.120470 33190735 PMC8750210

[16] Offeddu GS , Possenti L , Loessberg‐Zahl JT , et al. Application of transmural flow across in vitro microvasculature enables direct sampling of interstitial therapeutic molecule distribution. Small. 2019;15(46):1902393. doi:10.1002/smll.201902393 31497931

[17] Salipante PF , Hudson SD , Alimperti S . Blood vessel‐on‐a‐chip examines the biomechanics of microvasculature. Soft Matter. 2022;18(1):117‐125. doi:10.1039/D1SM01312B PMC9001019 34816867

[18] Bhatia SN , Ingber DE . Microfluidic organs‐on‐chips. Nat Biotechnol. 2014;32(8):760‐772. doi:10.1038/nbt.2989 25093883

[19] Zudaire E , Gambardella L , Kurcz C , Vermeren S . A computational tool for quantitative analysis of vascular networks. PLoS One. 2011;6(11):e27385. doi:10.1371/journal.pone.0027385 22110636 PMC3217985

[20] Vickerman MB , Keith PA , McKay TL , et al. VESGEN 2D: automated, user‐interactive software for quantification and mapping of angiogenic and lymphangiogenic trees and networks. Anat Rec. 2009;292(3):320‐332. doi:10.1002/ar.20862 PMC2880175 19248164

[21] Seaman ME , Peirce SM , Kelly K . Rapid analysis of vessel elements (RAVE): a tool for studying physiologic, pathologic and tumor angiogenesis. PLoS One. 2011;6(6):e20807. doi:10.1371/journal.pone.0020807 21694777 PMC3111429

[22] Corliss BA , Doty RW , Mathews C , Yates PA , Zhang T , Peirce SM . REAVER: a program for improved analysis of high‐resolution vascular network images. Microcirculation. 2020;27(5). doi:10.1111/micc.12618 PMC7507177 32173962

[23] Nicolas N , Roux E . 3D imaging and quantitative characterization of mouse capillary coronary network architecture. Biology. 2021;10(4):306. doi:10.3390/biology10040306 33917130 PMC8067837

[24] Bonda U , Jaeschke A , Lighterness A , et al. 3D quantification of vascular‐like structures in z stack confocal images. STAR Protoc. 2020;1(3):100180. doi:10.1016/j.xpro.2020.100180 33377074 PMC7757404

[25] Lang S , Müller B , Dominietto MD , et al. Three‐dimensional quantification of capillary networks in healthy and cancerous tissues of two mice. Microvasc Res. 2012;84(3):314‐322. doi:10.1016/j.mvr.2012.07.002 22796313

[26] Campisi M , Shin Y , Osaki T , Hajal C , Chiono V , Kamm RD . 3D self‐organized microvascular model of the human blood‐brain barrier with endothelial cells, pericytes and astrocytes. Biomaterials. 2018;180:117‐129. doi:10.1016/j.biomaterials.2018.07.014 30032046 PMC6201194

[27] Levick JR , Michel CC . Microvascular fluid exchange and the revised Starling principle. Cardiovasc Res. 2010;87(2):198‐210. doi:10.1093/cvr/cvq062 20200043

[28] Pries AR , Secomb TW . Blood flow in microvascular networks. Microcirculation. Elsevier; 2008:3‐36. doi:10.1016/B978-0-12-374530-9.00001-2

[29] Cassot F , Lauwers F , Fouard C , Prohaska S , Lauwers‐Cances V . A novel three‐dimensional computer‐assisted method for a quantitative study of microvascular networks of the human cerebral cortex. Microcirculation. 2006;13(1):1‐18. doi:10.1080/10739680500383407 16393942

[30] Secomb TW . Blood flow in the microcircaulation. Annu Rev Fluid Mech. 2017;49:443‐461. doi:10.1146/annurev-fluid-010816-060302

[31] Baxter LT , Jain RK . Transport of fluid and macromolecules in tumors. II. Role of heterogeneous perfusion and lymphatics. Microvasc Res. 1990;40(2):246‐263. doi:10.1016/0026-2862(90)90023-K 2250603

[32] Zhan W , Gedroyc W , Xu XY . The effect of tumour size on drug transport and uptake in 3‐D tumour models reconstructed from magnetic resonance images. PLoS One. 2017;12(2):e0172276. doi:10.1371/journal.pone.0172276 28212385 PMC5315397

[33] Offeddu GS , Haase K , Gillrie MR , et al. An on‐chip model of protein paracellular and transcellular permeability in the microcirculation. Biomaterials. 2019;212:115‐125. doi:10.1016/j.biomaterials.2019.05.022 31112823

[34] Colombo MV , Bersini S , Arrigoni C , et al. Engineering the early bone metastatic niche through human vascularized immuno bone minitissues. Biofabrication. 2021;13(3):035036. doi:10.1088/1758-5090/abefea 33735854

[35] Bersini S , Gilardi M , Arrigoni C , et al. Human in vitro 3D co‐culture model to engineer vascularized bone‐mimicking tissues combining computational tools and statistical experimental approach. Biomaterials. 2016;76:157‐172. doi:10.1016/j.biomaterials.2015.10.057 26524536

[36] Margolis EA , Cleveland DS , Kong YP , et al. Stromal cell identity modulates vascular morphogenesis in a microvasculature‐on‐a‐chip platform. Lab Chip. 2021;21(6):1150‐1163. doi:10.1039/d0lc01092h 33538719 PMC7990720

[37] Tarbell JM . Shear stress and the endothelial transport barrier. Cardiovasc Res. 2010;87(2):320‐330. doi:10.1093/cvr/cvq146 20543206 PMC2915475

[38] Wu D , Birukov K . Endothelial cell mechano‐metabolomic coupling to disease states in the lung microvasculature. Front Bioeng Biotechnol. 2019;7. doi:10.3389/fbioe.2019.00172 PMC6658821 31380363

[39] Schmid F , Barrett MJP , Obrist D , Weber B , Jenny P . Red blood cells stabilize flow in brain microvascular networks. PLoS Comput Biol. 2019;15(8):e1007231. doi:10.1371/journal.pcbi.1007231 31469820 PMC6750893

[40] Lowerison MR , Sekaran NVC , Zhang W , et al. Aging‐related cerebral microvascular changes visualized using ultrasound localization microscopy in the living mouse. Sci Rep. 2022;12(1):619. doi:10.1038/s41598-021-04712-8 35022482 PMC8755738

[41] Clendenon SG , Fu X , Von Hoene RA , et al. A simple automated method for continuous fieldwise measurement of microvascular hemodynamics. Microvasc Res. 2019;123:7‐13. doi:10.1016/j.mvr.2018.11.010 30502365 PMC6379124

[42] Balogh P , Bagchi P . Three‐dimensional distribution of wall shear stress and its gradient in red cell‐resolved computational modeling of blood flow in in vivo‐like microvascular networks. Physiol Rep. 2019;7(9):e14067. doi:10.14814/phy2.14067 31062494 PMC6503071

[43] Haslam M , Zamir M . Pulsatile flow in tubes of elliptic cross sections. Ann Biomed Eng. 1998;26(5):780‐787. doi:10.1114/1.106. 9779950

[44] Hogan B , Shen Z , Zhang H , Misbah C , Barakat AI . Shear stress in the microvasculature: influence of red blood cell morphology and endothelial wall undulation. Biomech Model Mechanobiol. 2019;18(4):1095‐1109. doi:10.1007/s10237-019-01130-8 30840162

[45] The MathWorks Inc. Matlab. Published online 2021.

[46] Chan TF , Vese LA . Active contours without edges. IEEE Trans Image Process. 2001;10(6):266‐277. doi:10.1016/j.mcm.2011.11.014 18249617

[47] Yu G , Li P , Miao YL , Bian ZZ . Multiscale active contour model for vessel segmentation. J Med Eng Technol. 2008;32(1):1‐9. doi:10.1080/03091900600700798 18183515

[48] Oh J , Martin DR , Hu X . Partitioned edge‐function‐scaled region‐based active contour (p‐ESRAC): automated liver segmentation in multiphase contrast‐enhanced MRI. Med Phys. 2014;41(4):1‐11. doi:10.1118/1.4867865 24694145

[49] Pissas T , Bloch E , Cardoso MJ , et al. Deep iterative vessel segmentation in OCT angiography. Biomed Opt Express. 2020;11(5):2490‐2510. doi:10.1364/boe.384919 32499939 PMC7249805

[50] Jaworek‐Korjakowska J . A deep learning approach to vascular structure segmentation in dermoscopy colour images. Biomed Res Int. 2018;2018:5049390. doi:10.1155/2018/5049390 30515404 PMC6236870

[51] Livne M , Rieger J , Aydin OU , et al. A U‐net deep learning framework for high performance vessel segmentation in patients with cerebrovascular disease. Front Neurosci. 2019;13:97. doi:10.3389/fnins.2019.00097 30872986 PMC6403177

[52] Ronneberger O , Fischer P , Brox T . U‐net: convolutional networks for biomedical image segmentation. Lecture Notes in Computer Science (Including Subseries Lecture Notes in Artificial Intelligence and Lecture Notes in Bioinformatics) 2015;9351:234–241. doi:10.1007/978-3-319-24574-4_28

[53] Lee TC , Kashyap RL , Chu CN . Building skeleton models via 3‐D medial surface/axis thinning algorithms. CVGIP: Graph Models Image Process. 1994;56(6):462‐478. doi:10.1006/cgip.1994.1042

[54] Kollmannsberger P , Kerschnitzki M , Repp F , Wagermaier W , Weinkamer R , Fratzl P . The small world of osteocytes: connectomics of the lacuno‐canalicular network in bone. New J Phys. 2017;19(7). doi:10.1088/1367-2630/aa764b

[55] Offeddu GS , Hajal C , Foley CR , et al. The cancer glycocalyx mediates intravascular adhesion and extravasation during metastatic dissemination. Commun Biol. 2021;4(1):255. doi:10.1038/s42003-021-01774-2 33637851 PMC7910477

[56] Possenti L , di Gregorio S , Gerosa FM , et al. A computational model for microcirculation including Fahraeus‐Lindqvist effect, plasma skimming and fluid exchange with the tissue interstitium. Int J Numer Method Biomed Eng. 2019;35(3):e3165. doi:10.1002/cnm.3165 30358172

