# Supplementary material for: A three‐dimensional method for morphological analysis and flow velocity estimation in microvasculature on‐a‐chip
Source: Bioeng Transl Med. 2023 Jun 11;8(5):e10557. doi: 10.1002/btm2.10557 (PMC10487341; doi:10.1002/btm2.10557)
Supplement: Supplementary file 1 — Figure S1: Orientation of the major diameter of the fitted elliptical cross‐section of the vessels on a single image (a). Cross‐section of a portion of the network obtained with deep learning (b) with true positive (TP—green), false positive (FP—blue), false‐negative (FN—red), and true negative (TN—black). (c) Eccentricity map on a network. (d) Drawings showing the difference between 2D and 3D data to estimate the network lateral area. The top view depicts 2D analysis. The mid‐level represents the lateral area computation based on the 2D radius, that is, implying a circular section. The bottom view shows results from 3D images, reporting a noncircular cross‐section. Figure S2: Computational analysis of the radius uncertainty effect on velocity and WSS estimates. We analyzed a single vessel (a) and a simplified network of eight vessels (b). (c) With reference to a single vessel, we set a nominal radius of 15 μm and a radius uncertainty of 1.6 μm. We then created 106 scenarios with a radius from a normal distribution with a mean of 15 μm and a standard deviation of 1.6 μm. We fix the length (100 μm) and the pressure difference (to ensure 500 μm/s when choosing r = 15 μm). We report the resulting velocity and WSS distributions. Interestingly, WSSs are normally distributed (avg: 133 mPa, SD: 14 mPa), while the velocity is not (but its square root follows a Gaussian distribution). (d) Median values for radius, velocity, and WSS over the eight‐vessel network considering the same nominal radius for each vessel. Data are not normally distributed anymore. The median value variations are still comparable to the single branch case. (e) Results from the eight‐vessels network with nominal radius values randomly chosen among the permutation of the set {13, 14, 14, 15, 15, 16, 16, 17} μm. Then, we applied similar methods to compute 106 cases with 1.6 μm radius uncertainty for each vessel. Ranges of variation for median values are still comparable to the previous case. [file BTM2-8-e10557-s001.docx]

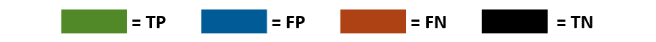


**Figure S1:** Orientation of the major diameter of the fitted elliptical cross-section of the vessels on a single image (a). Cross-section of a portion of the network obtained with deep learning (b) with true positive (TP - green), false positive (FP - blue), false-negative (FN- red), and true negative (TN – black). (c) Eccentricity map on a network. (d) Drawings showing the difference between 2D and 3D data to estimate the network lateral area. The top view depicts 2D analysis. The mid-level represents the lateral area computation based on the 2D radius, i.e., implying a circular section. The bottom view shows results from 3D images, reporting a non-circular cross-section.

**Figure S2:** Computational analysis of the radius uncertainty effect on velocity and WSS estimates. We analyzed a single vessel (a) and a simplified network of eight vessels (b). (c) With reference to a single vessel, we set a nominal radius of 15 µm and a radius uncertainty of 1.6 µm. We then created 106 scenarios with a radius from a normal distribution with a mean of 15 µm and a standard deviation of 1.6 µm. We fix the length (100 µm) and the pressure difference (to ensure 500 µm/s when choosing r = 15 µm). We report the resulting velocity and WSS distributions. Interestingly, WSSs are normally distributed (avg: 133 mPa, SD: 14 mPa), while the velocity is not (but its square root follows a Gaussian distribution). (d) Median values for radius, velocity, and WSS over the eight-vessel network considering the same nominal radius for each vessel. Data is not normally distributed anymore. The median value variations are still comparable to the single branch case. (e) Results from the eight-vessels network with nominal radius values randomly chosen among the permutation of the set {13, 14, 14, 15, 15, 16, 16, 17} µm. Then, we applied similar methods to compute 106 cases with 1.6 µm radius uncertainty for each vessel. Ranges of variation for median values are still comparable to the previous case.
